# Case of Disease of the Heart; with the Post-Mortem Appearances

**Published:** 1825-06

**Authors:** William Baker


**Art. VI.**—*Case of Disease of the Heart; with the Post-Mortem Appearances.* By William Baker, M.D. &c.

I forward to you the narrative of a case taken from my notes, which, from the severity of the symptoms, its fatal termination, and the nature of the disease found on examining the body, you may, perhaps, think worthy of publication in your Journal.

41, *Conduit-street, Hanover-square; April*, 1825.

A. K. aged forty-seven; married woman.

January 25th, 1825.—Complains of some difficulty of breathing, but chiefly of great commotion at the heart, and down the left side of the abdomen. Has been thus affected for twenty-three days; and believes her disorder to have been brought on by waiting long in a cold and damp room, she being then warm from previous exercise. Never had rheumatism, but has enjoyed continued good health, with the exception of an attack a year since, similar to the present; which, however, lasted only a few days, and subsided without medical assistance. Menstruation ceased eight months ago. She is the mother of eleven children, nine of whom are living; and, in addition to the care of a large family, has experienced much domestic anxiety.

*Present state.*—Sleep broken; pulse 152, very irregular, and intermitting. Heart beating with great violence, quickness, and irregularity; its action not corresponding with the pulse at the wrist. With the help of the stethoscope, the auricle was heard at times performing two or three contractions, whilst the ventricle contracted only once; and the next moment the reverse would often take place. In short, the greatest irregularity existed, and with frequent change from one kind of error to another. The respiration was rather hurried, as if subsiding after running.

I ordered her to be bled from the arm to the extent of eight ounces, and to take a laxative mixture.

Jan. 26th.—The heart's impulse rather less, but the pulse very frequent, and more irregular than yesterday, so that to count it at the heart or wrist was impossible. No agreement between the heart and the pulse at the wrist.

She was again bled from the arm to the amount of fifteen ounces. She passed a good night, and continued relieved until—

February 9.—The difficulty in respiring was now increased. She slept but little. The pulse was irregular, and the bowels had become very costive. On examination with the stethoscope, there was much mucous rattle at the upper part of the right lung, and a little in the left.

A blister to the chest, and aperient medicine, gave her great relief: the former did not rise well. The heart's action became more moderate from this time; but the pulse continued frequent, varying between 125 to 140 in the minute. The respiration was more free, and the mucous rattle diminished.

Feb. 17th.—She had now become worse. Her pulse was very quick and irregular, but not intermitting as formerly. The beat of the heart was very strong. At times she coughed violently; and the difficulty of breathing was greater, as also was the mucous rattle in the right lung. Her legs, during the day, were anasarcous.

From this time she got progressively worse. Her respiration was still more imperfect and laborious, and the distress and anxiety were extremely great. Blood was again taken away by cupping on the chest, and blisters repeated, but without any essential benefit.

Medicine was given to relieve urgent symptoms, both now and in earlier stages of this case, which I have purposely omitted: it being intended only to abate fever, procure sleep, or meet any other indication then existing, but not expected to remove organic disease.

March 1st.—The respiration was now almost extinguished; the countenance livid, with profuse perspiration on the face, neck, and chest. The expectoration was rather bloody.

March 3d.—She died this evening.

*Examination, forty hours after death.*

The body was of middle stature, and its condition inclining to fat. The legs were anasarcous, particularly the right.

*Thorax.*—The chest was capacious. The lungs inflated, and crepitated when pressed between the thumb and finger. The right lung appeared healthy at its upper part, but the middle and lower lobes had a marbled appearance, caused by portions of lung seen through the pleura, of a dark purple colour, for the most part oval or rounded, and having a distinctly defined edge. These dark portions of lung, when cut into, were solid; and, in the lower and middle lobes of this lung, the change here described had taken place to a great extent. This alteration of structure appeared to have been produced by hemorrhage into the substance of the lung, which had caused ecchymosis around the ruptured vessel, rendering that portion of lung solid, and thereby obliterating the air-cells. Such was the explanation offered by my friend Dr. Urquhart, who assisted me in examining the body, and who has studied the morbid anatomy of the lungs under the able instruction of M. Laennec, in Paris. A large part of the lower lobe was also hepatised, but had not much firmness, nor any defined boundary, similar to that above mentioned.

The right cavity of the chest contained about a pint and a half of serous fluid, with a few flakes of coagulated lymph.

A part of the pleura covering the diaphragm had its vessels beautifully injected by inflammation, and the adjoining pleura costalis was slightly inflamed.

In the left side of the chest, there were about six ounces of serum. The lung here was sound, except a few spots, which were dark coloured and solid, and resembled the disease in the right lung.

*Heart.*—In the pericardium there was found rather more than an ounce of serum. The heart was large. Its lower third being cut off transversely, showed both ventricles and both auricles distended with dark coagulated blood; but there was not any separation of the coagulable lymph, so often found in jelly-like masses in the heart: except, indeed, in the right auricle, where that had occurred to a small extent. The walls of the left ventricle were rather thicker than usual: those of the right not decidedly so; but its structure a little softer than natural, and it was somewhat dilated. The right auricle was considerably dilated. The greatest morbid change, however, was in the state of the tricuspid valve, which was thickened and of a cartilaginous appearance and hardness, but without any bony deposit; and the auriculo-ventricular opening was not diminished in extent. The valves of the pulmonary artery were sound. The left ventricle was also dilated; and the mitral valve was found in a state similar to the tricuspid; and the valves of the aorta were likewise thickened, opaque, and cartilaginous.

*Abdomen.*—There was a good deal of serum here, but the viscera were healthy.

*Remarks on the foregoing Case.*

The disease existing in the lungs, and that in the heart, cannot, in my opinion, be regarded as holding any relation to each other, as cause and effect. The diseased state of the auriculo-ventricular valves had probably existed for a long period, and, as it appears from the case, without any inconvenience, until a state of congestion and impeded circulation in the lungs, brought on by exposure to cold, occasioned in a heart previously so diseased the great irregularity and disturbance detailed in the symptoms. The direct effect of the defective action of the tricuspid valve would be to allow a reflux of the blood into the auricle during the systole of the ventricle, which accounts for the dilated state of the former; but such a derangement must have greatly lessened the impetus with which the blood would be thrown on the lungs, as compared with the action of a healthy heart: it, therefore, could not have contributed to produce the disease existing there. And, on the other hand, it is impossible to imagine the morbid state of the lungs was antecedent to, and the cause of, organic disease of the heart; because such a state of lungs would have been incompatible with the good health which this patient had experienced, with only one interruption of a few days: nor do we find such disease of the heart accompanying an impervious state of the lungs, even in cases where it is more extensive than in the present.

I should have anticipated a pulsation in the veins of the neck, from the imperfect action of the tricuspid valve; but it did not occur.

